# Hypolipidemic and Hypoglycaemic Effect of Wholemeal Bread with Amaranth (*Amaranthus dubius* Mart. ex Thell.) on Sprague Dawley Rats †

**DOI:** 10.3390/foods9060707

**Published:** 2020-06-01

**Authors:** Adriana Beatriz Sánchez-Urdaneta, Keyla Carolina Montero-Quintero, Pedro González-Redondo, Edgar Molina, Belkys Bracho-Bravo, Rafael Moreno-Rojas

**Affiliations:** 1 Departamento de Botánica, Facultad de Agronomía, Universidad del Zulia, Maracaibo 4001, Venezuela; usanchez@fa.luz.edu.ve; 2 Facultad de Ingeniería Agronómica, Universidad Técnica de Manabí, Manabí 130105, Ecuador; 3 Departamento de Química, Facultad de Humanidades y Educación, Universidad del Zulia, Maracaibo 4001, Venezuela; keylamq@gmail.com (K.C.M.-Q.); molinaed01@gmail.com (E.M.); 4 Departamento de Ciencias Agroforestales, Escuela Técnica Superior de Ingeniería Agronómica, Universidad de Sevilla, 41013 Sevilla, Spain; 5 Departamento de Estadística, Facultad de Agronomía, Universidad del Zulia, Maracaibo 4001, Venezuela; bjbrachob@fa.luz.edu.ve; 6 Departamento de Bromatología y Tecnología de los Alimentos, Universidad de Córdoba, 14071 Córdoba, Spain; rafael.moreno@uco.es

**Keywords:** *Amaranthus dubius*, bread, hyperlipidaemia, hypoglycaemia, pseudocereal, rat

## Abstract

The effect of consuming breads made with wheat flour and Amaranth (*Amaranthus dubius* Mart. ex Thell.) wholemeal flour on Sprague Dawley rats with hyperlipidaemia and hyperglycaemia induced through feeding was studied. Four diets were provided: control (CD: Ratarina^®^), commercial bread (CBD), bread with 100 g·kg^−1^ (ABD10) and 200 g·kg^−1^ (ABD20) amaranth flour. Zoometric and blood chemistry parameters were measured before and after consuming the diets. A completely random factorial design of 2 × 4 × 2 was used. The factors were blood lipids and glucose level (normal, N and elevated, E), diet (CD, CBD, ABD10 and ABD20) and sex (female, F and male, M). The rats consuming ABD10 and ABD20 diets presented the lowest glucose values, although with no differences (*p* > 0.05) between the groups of elevated blood lipids and glucose rats (E). Triglyceride concentrations decreased in ABD10 and ABD20 treatments in comparison with CD, elevated blood lipids and glucose (E) rats, while ABD10 rats showed lower total cholesterol level than normal (N) rats. The high-density lipoprotein cholesterol values increased in the ABD10 and ABD20 groups (*p* < 0.05), while it did lower for very low-density lipoprotein cholesterol and cardiac risk index (*p* < 0.05). In ABD10 and ABD20 treatments, the abdominal circumference decreased in both sexes (*p* < 0.05) between weeks 23 and 31. In conclusion, consumption of bread with amaranth improved lipid profiles of rats and could help to prevent metabolic and cardiovascular diseases.

## 1. Introduction

The main causes of morbidity and mortality in Western countries, Asia and the Pacific are cardiovascular disease and diabetes [1,2]. The main risk factors for atherosclerosis and diabetes are hyperglycaemia and hyperlipidaemia [3,4], which are directly related to diet and lifestyle [2,5,6].

Western-type diets, rich in saturated fats, simple carbohydrates and low in fibre, are recognized as a risk factor for atherosclerosis and diabetes [2]. On the other hand, studies confirm that a larger amount of fibre in the diet causes a lower incidence of these diseases [7,8,9]. Therefore, consuming cereals and pseudocereals helps to prevent diseases associated with metabolic syndromes [10,11,12].

There is a worldwide need to seek alternative foods in order to improve the nutritional status of the population and to prevent the incidence of metabolic diseases associated with the intake of certain foods. Seeds and shoots are an excellent example of functional foods whose consumption has increased among people interested in improving and maintaining good health because their components decrease the risk of various diseases and/or promote beneficial health effects [13]. However, most of the recently published research has focused mainly on the study of typical, commercially available shoots. Nevertheless, in recent decades, the use of seeds, amaranth sprouts, and other nontraditional vegetables has increased. Not only this increase has occurred in the great majority of people, but also in vegans, vegetarians, celiacs, and those who are allergic to some commonly consumed vegetables [14,15,16,17].

*Amaranthus* genus includes a diversity of plants adapted to a wide variety of environments and tolerant to adverse conditions, mainly dry soils and high temperatures [18,19]. It grows wild and as a weed in several subsistence crops [20]. In several tropical countries, such as Venezuela, there are about 12 widely distributed species, but *Amaranthus dubius* Mart. ex Thell. is a one of the most numerous; it is native to South America and was introduced to Asia, Africa and Europe [20,21,22,23]. It presents the growing characteristics necessary for exploitation as a crop, and due to its nutritional and agronomic characteristics, it was included in the ancestral foods rescue program in Venezuela and has, since 2005, been considered a wild species with a potential for cultivation [21].

Cultivated *A. dubius* has a high concentration of proteins and minerals [24], low concentrations of toxic and antinutritional factors [25], and does not contain heavy metals such as Cd and Pb [26]. This makes it of great nutritional interest because it could become a means of improving nutrition and maintaining and strengthening the human immune system. It could also be used in the preparation of gluten-free products, i.e., functional foods for use in treating certain diseases associated with food consumption [27,28]. The nutritional and agronomic characteristics of amaranth make it a plant of potential interest for use in the human food and animal feed industries [29,30,31].

Bread is a staple component of the diet in many parts of the world, and because of its high carbohydrate content, it contributes about 50% of the diet’s energy input [32]. Digestion of the carbohydrates in bread affects glucose absorption and the metabolic glucose and lipids regulatory mechanisms [33,34].

Many studies have aimed at improving the nutritional value of bread with functional ingredients. Several of them recommend adding mixtures of seeds, the grains of other cereals or nuts [35]. In recent times, grain of several amaranth species has gained renewed interest as a functional ingredient, especially in bread making and bakery processes, since it is highly versatile for transformation and industrialization [36,37,38,39,40].

Previous studies report improvement in the lipid profile and glycaemic index of human and animals consuming food including grain of several amaranth species [28,41]. However, there are no previous reports on the hypoglycaemic and hypolipidemic effects of bread made with the addition of *A. dubius* flour. We hypothesise that such flour could be an alternative food for patients with metabolic syndromes or for preventing of related diseases in healthy individuals. Therefore, the present study aimed to analyse the metabolic effects on glycaemic and lipidemic levels of consumption of bread with added amaranth (*A. dubius*) flour in female and male Sprague Dawley rats with high sucrose consumption.

## 2. Materials and Methods

### 2.1. Obtaining Amaranth Flour

Samples of *A. dubius* were obtained from an experimental sowing located on a farm in the Santa Rita municipality, Zulia State, Venezuela (geographic coordinates: 10°37’ N, 71°08’ W). The soil was prepared with a harrow and fertilized with organic matter (plant layer and poultry manure). The panicles were dehydrated in a stove (50 to 60 °C for 40 h) with rotation and constant aeration. They were subsequently ground, sifted with particle size ≤0.5 mm (Retsch Muhle Dietz, LB1-27^®^, Haan, Germany) and stored in polyethylene containers with a hermetic lid, covered with a fabric bag and kept in wooden cases at temperatures of ≤20 °C until being used in bread preparation [42].

### 2.2. Preparation of the Breads

Breads with amaranth (100 and 200 g kg^−1^) were prepared mixing wheat flour and amaranth wholemeal flour and subsequently adding this mixture to the other components (g kg^−1^): 15 salt, 60 sugar, 50 fat, 50 yeast and 550 water. The dough was mixed and kneaded for 15 min (Boia^®^ 20 L, Carrizal, Venezuela) and subsequently kneaded manually. It was then weighed and placed in a sandwich bread mould. Fermentation occurred at 30–35 °C for 180 min and the bread was baked at 160–170 °C for 1 h 10 min [43].

### 2.3. Experimental Diets 

Four diets were prepared: control diet (CD), a specific food for rodent consumption (Ratarina^®^, Protinal, Valencia, Venezuela); commercial bread diet (CBD), by acquiring a special commercial bread for hypertensive, diabetic and obese people (Bimbo^®^, Guarenas, Venezuela); and diet breads with 100 g kg^−1^ (ABD10) and 200 g kg^−1^ amaranth (ABD20). To prepare the ABD10, ABD20 and CBD diets, the loaves were cut up and dried at 60 °C for 48 h. Then they were subsequently ground for use in the preparation of pellets (2 mm diameter × 2 mm length) using a meat mill adapted by means of a special disc, and subsequently baked (180 °C for 20 min). 

Ingredients and proximal composition of the experimental diets are shown in Table 1. The Association of Official Analytical Chemists (AOAC) International [44] procedures were used to determine dry matter (DM, method 934.01), crude protein (CP, method 976.05, Kjeldahl: N × 6.25) and ether extract (EE, method 2003.05). Crude fibre (CF) and ash were determined according to AOAC International procedures [45] (methods 962.09 and 942.05, respectively). Nitrogen free extract (NFE) and total digestible nutrients (TDN) were calculated as follows:

NFE (g kg^−1^) = 1000 − (g kg^−1^ CP + g kg^−1^ CF + g kg^−1^ EE + g kg^−1^ Ash). (1)

TDN (g kg^−1^) = g kg^−1^ digestible CF + g kg^−1^ digestible NFE + g kg^−1^ digestible CP + 2.25 × g kg^−1^ digestible EE. (2)

The energy contents of the experimental diets were estimated as follows, using the Atwater general factor system [46]:

Energy (kcal kg^−1^) = (g kg^−1^ CP × 4) + (g kg^−1^ EE × 9) + (g kg^−1^ Total Carbohydrate × 4) with total carbohydrate obtained as CF + NFE contents.

### 2.4. Animals and Housing Conditions

A total of 60 Sprague Dawley rats (30 males and 30 females) aged 21 days, supplied by the vivarium at the Central-Western Lisandro Alvarado University, Barquisimeto, Lara State, Venezuela were used. The animals were housed in individual wire mesh cages measuring 25  ×  25  ×  25 cm, maintained in a 12:12 h light:dark cycle at 25 °C, and were fed the CD and water ad libitum during a 7-day adaptation period.

Rat management and handling, as well as the experimental protocol, were performed according to the Directive 2010/63/EU on the protection of animals used for scientific purposes [47].

### 2.5. Induction of Hyperglycaemia and Hyperlipidaemia

Hyperglycaemia and hyperlipidaemia were induced in 48 rats (24 males and 24 females; E: elevated blood lipids and glucose [hyperlipidaemic and hyperglycaemic] animals) via ingestion ad libitum of CD and an aqueous solution of 300 g L^−1^ sucrose during 23 weeks (weeks 1 to 23 of the trial) [48,49]. The remaining 12 rats (6 males and 6 females) were maintained at their normal blood lipids and glucose levels (N: normal animals) with an ad libitum intake of CD and drinking water.

Subsequently, 10 animals (five males and five females) were randomly selected from each group (E and N), and their zoometric parameters were measured: body weight, body length, body mass index (BMI) and abdominal circumference (AC). Blood samples were taken from the coccygeal vein, in rats previously anesthetized with ether [50] for determinations of blood chemistry.

### 2.6. Experimental Diets Assay

Forty-eight (48) animals with elevated blood lipids and glucose levels (E: hyperlipidaemic and hyperglycaemic) were divided into four groups of 12 animals each (six females and six males) according to the experimental diets: CD, CBD, ABD10 and ABD20. All groups consumed ad libitum an aqueous solution of 30% sucrose and the assigned specific diet for 8 weeks (weeks 23 to 31 of the trial). The group of normal animals (N: six males and six females) received CD and drinking water ad libitum. At the end of the experimental period, the zoometric parameters were measured again, and samples were taken for blood chemistry analysis, following the procedure described above. The animals were then euthanised by cervical dislocation, after being previously anesthetized with ether in a glass bell.

### 2.7. Biochemical Analysis of Serum

Blood samples were centrifuged at 2000 rpm for 5 min to obtain serum, which was then frozen at −20 °C for later analysis. Glucose (GLU), triglycerides (TG), total cholesterol (TC), high-density lipoprotein cholesterol (HDL-C) and total protein (TP) were analysed. In the determination of HDL-C, a kit (HDL Cholesterol^®^, ref. 10018, Human Gesellschaft für Biochemica und Diagnostica GmbH, Wiesbaden, Germany) was used. Commercial test kits (Wiener Lab, Rosario, Argentina) were used to determine TG, TC and GLU by colorimetry. Total protein determination was performed by a Proti 2^®^ kit (Wiener Lab, Rosario, Argentina).

The following indices were calculated: TC/LDL-C, HDL-C/LDL-C. Very low-density lipoprotein cholesterol (VLDL-C), low-density lipoprotein cholesterol (LDL-C), atherogenic index and cardiac risk factor were calculated using the following formulas [51,52,53]:

VLDL-C = TG/5.

LDL-C = TC − HDL-C − (TG/5).

Atherogenic index = (TC − HDL-C)/HDL-C.

Cardiac risk factor = TC/HDL-C.

### 2.8. Statistical Analysis

The data were analysed using a randomized 2 × 4 × 2 factorial design, and the research factors were the blood lipids and glucose level of the animal (normal, N and elevated, E), diet (CD, CBD, ABD10, and ABD20), and sex of rats (male, M and female, F). The simple effects of the treatments were analysed with Tukey’s multiple comparison tests and the interaction of treatments with the generalised linear model (GLM) procedure with four replications and three subsamples. The body weight variation in the experimental groups during the study period was analysed by repeated measures in time analysis of variance through the mixed linear model (MIXED) procedure of Statistical Analysis Software (SAS), and then by selecting the second-degree polynomial models that best explained the behaviour of this variable over time. The statistical analyses were performed using SAS v.9.1.3 (SAS Institute Inc., Cary, NC, USA).

## 3. Results

### 3.1. Induction of the Hyperglycaemia and Hyperlipidaemia

Table 2 shows the results of the zoometric and biochemical parameters evaluated during induction tests of hyperglycaemia and hyperlipidaemia in rats. There were no significant differences in the zoometric parameters between rats consuming sucrose water for 23 weeks and control rats (*p* > 0.05). In the biochemical profile, significant differences between both groups were observed in the GLU (*p* < 0.05), TC (*p* < 0.05), TG (*p* < 0.001), and LDL-C (*p* < 0.001) values, which increased in rats consuming sucrose water. This group displayed a reduction in VLDL-C (*p* < 0.001), TC/LDL-C (*p* < 0.01), HDL-C/LDL-C (*p* < 0.01) and TP (*p* < 0.05) after the consumption of sucrose water. On the other hand, no significant differences were found in HDL-C and TC/HDL-C (*p* > 0.05).

### 3.2. Effect of Diets Consumption on Zoometric Parameters

No statistical differences (*p* > 0.05) were observed for body weight, body length and body mass index (BMI) when comparing (by pairs between them) the different experimental diets consumed for 8 weeks (weeks 23 to 31 of age).

Table 3 shows the results of the abdominal circumference (AC) due to the consumption of the experimental diets for 8 weeks (weeks 23 to 31 of age). At the starting of the consumption of experimental diets (week 23 of age), rats of both sexes did not show differences (*p* > 0.05) in AC for all comparisons of pairs between the experimental diets.

After 8 weeks of consumption of the experimental diets (week 32 of age), the rats of both sexes which consumed the diets with amaranth (ABD10(E) and ABD20(E) diets) showed lower AC than rats consuming CD(N), CD(E) and CBD(E) diets (*p* < 0.05), when compared by pairs (Table 3). Conversely, AC did not vary between CD(N), CD(E) and CBD(E) treatments (*p* > 0.05). Moreover, no difference was observed after 8 weeks of consumption between ABD10(E) and ABD20(E) rats (*p* > 0.05).

**Figure 1:**
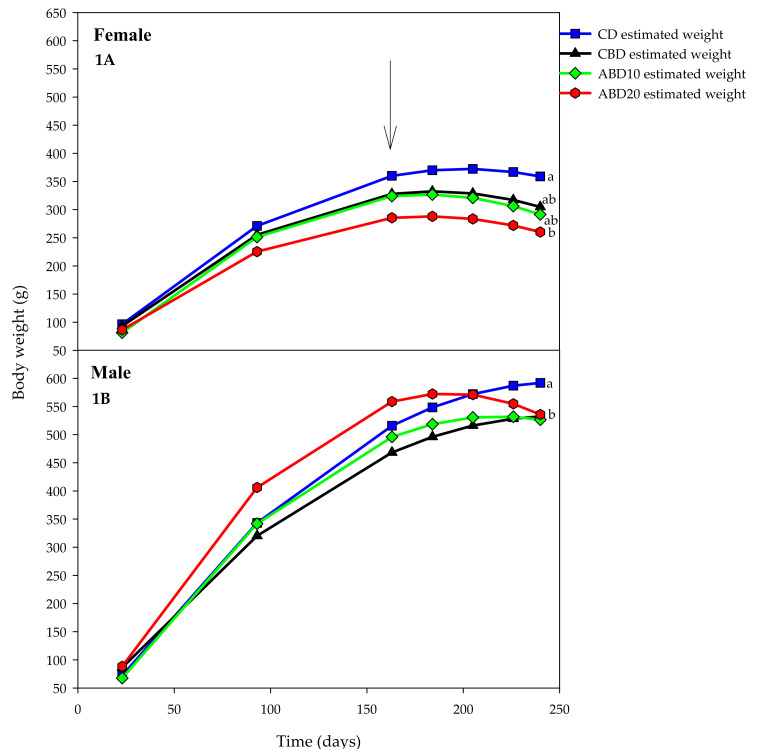
Body weights (mean) of control diet (CD(E), square, blue), control bread diet (CBD(E), triangle, black), diet bread with 100 g·kg^−1^ amaranth (ABD10(E), diamond, green) and diet bread with 200 g·kg^−1^ amaranth (ABD20(E), circle, red) rats during 240 days of the protocol. (**1A**): female rats; (**1B**): male rats. E: elevated blood lipids and glucose levels. The arrow indicates the start of experimental diets consumption. Body weights at the end of the trial showing different letters are significantly different (*p* < 0.05).

The variation in body weight of the rats during the study is shown in Figure 1 (weeks 1 to 31). The female and male rats had different weight increases (*p* < 0.01) even when the initial weights were similar. During the hyperglycaemia and hyperlipidaemia induction period, the behaviour of the weight gain in the male and female rats were comparable (Figure 1A,B). However, from day 163 (the start of the experimental diets) up to day 240 of age, variable behaviour between sexes was observed. In CD(E) treatments, body weights continued to increase until the end of the experimental period, whereas groups CBD(E), ABD10(E) and ABD20(E) rats began their weight loss as of day 184, a trend that was maintained until the end of the trial.

Different growth (*p* < 0.01) was found for female rats in the CD(E) and ABD20(E) treatments, with the other treatments being intermediate between them. It stands out that ABD20(E) rats’ body weight was the lowest during the study, although it did not differ from that of CBD(E) and ABD10(E) treatments (Figure 1A).

The growth pattern of male rats (Figure 1B) was slightly different from of females. Males fed the ABD20(E) diet presented the highest body weight until day 184, although it decreased thereafter, remaining similar to that of CBD(E) and ABD10(E) treatments at 240 days of age, without differences among them (*p* > 0.05). At 24 days of age, male rats of the CD(E) treatment reached a higher weight than those of the other three treatments (*p* < 0.001; Figure 1B).

### 3.3. Effect of Diets Consumption on Serum Biochemical Parameters

Table 4, Table 5 and Table 6 show the serum biochemical parameters of animals after 8 weeks (weeks 23 to 31 of age) being fed the experimental diets. Significant differences (*p* < 0.05) among treatments were found when comparing pairs for most of the serum biochemical parameters evaluated, except for TC/LDL-C and HDL-C/LDL-C (data not showed for these two parameters).

Higher GLU values were found in rats with elevated blood lipids and glucose levels (E) when compared with rats maintained normal (N) (Table 4). ABD10(E) did not display any statistical differences with respect to ABD20(E) rats (*p* > 0.05). Females fed the CBD(E) diet showed higher GLU levels than rats consuming CD(N) diet (*p* < 0.01), while in males, higher GLU values were found for CD(E) (*p* < 0.01), CBD(E) (*p* < 0.05) and ABD10(E) (*p* < 0.05) treatments with respect to the CD(N) diet (Table 4).

The lowest TC and TG concentrations were found in ABD10(E) and ABD20(E) treatments (Table 4). No statistical differences (*p* > 0.05) were found for the females in TC values between experimental diets compared by pairs, while the CD(N) males showed higher TC levels than ABD10(E) ones (*p* < 0.05). On the other hand, and regardless of sex, the TG values were lower (*p* < 0.05) in ABD10(E) and ABD20(E) rats than in CD(E) treatment. Male CD(E) rats showed higher TG levels than CD(N) ones (*p* < 0.001).

Regardless of sex, the highest high-density lipoprotein cholesterol levels were found for the ABD10(E) and ABD20(E) treatments (range 47.75–47.80 and 41.00–51.15 mg dL^−1^ for females and males, respectively), in relation to CD(N), CD(E) and CBD(E) experimental groups (*p* < 0.05) (Table 5). No statistical differences were seen between ABD10(E) and ABD20(E) (*p* > 0.05). However, no difference in HDL-C was observed for males between ABD20(E) and CD(E) rats.

Low-density lipoprotein cholesterol was low in ABD10(E) and ABD20(E) treatments (with no difference between them, in both sexes; *p* > 0.05), the lowest concentration being found in ABD10(E) males (Table 5). Male rats fed ABD10(E) and ABD20(E) diets showed lower LDL-C values than CD(N) rats (*p* < 0.05).

Regardless of sex, VLDL-C was lower in ABD10(E) and ABD20(E) treatments (with no difference between them; *p* < 0.05) in comparison to CD(E) rats (Table 5). Male rats fed ABD20(E) diet showed lower VLDL-C values than CBD(E) rats (*p* < 0.05).

The lower risk of suffering from some cardiovascular disease (cardiac risk factor) and the lowest atherogenic index were presented by ABD10(E) and ABD20(E) treatments both in females and males, in comparison to CD(N), CD(E) and CBD(E) diets (*p* > 0.05) (Table 6). No statistical differences were observed between ABD10(E) and ABD20(E) rats for both indices (*p* > 0.05).

## 4. Discussion

Consumption of breads including *A. dubius* flour at 100 and 200 g·kg^−1^ by hyperlipidaemic and hyperglycaemic rats permitted them to kept low serum GLU, and to reduce TC, TG and VLDL-C, while increased their HDL-C levels, improving their lipid profile. With these amaranth diets, the abdominal circumference decreased.

The induction of hyperglycaemia and hyperlipidaemia to rats via the ingestion of 30% sucrose solution m/v for 23 weeks was achieved satisfactorily, causing them an increase in GLU, TG and TC levels. This agrees previous reports demonstrating that supplementation with high sucrose concentrations is effective for induction of metabolic syndrome in laboratory rats, causing increased body weight, insulin, triacylglycerol, TC, LDL-C, and free fatty acids, among other anomalies [49,54,55]. The weight gain in the rats during the induction of the hyperglycaemia and hyperlipidaemia was similar between the groups, consuming or not consuming sucrose water, which is consistent with what was found by Girard et al. [56] in rats fed 60% fructose in their diets.

There are few research works on the use of vegetables and other raw materials alternative to cereal grains in bread baking and their effects on the prevention and treatment of metabolic and cardiovascular diseases. Most assays have studied the effects of the consumption of certain vegetables, their extracts and fungus [57,58,59].

The zoometric parameters are mostly used to evaluate the degree of obesity are AC and BMI, which have been associated with the risk of cardiovascular diseases [60], and mortality in patients with type 2 diabetes [61], although these are rarely assessed in assays with rats. The AC decreased in ABD10(E) and ABD20(E) rats after 8 weeks of consumption of the these diets including *A. dubius*, which is a relevant indicator of the reduction in visceral fat, which at the same time reduces the risk of metabolic syndrome [62], possibly influenced by the high fibre content in *A. dubius* [24,42,63]. It is well known that fibre increases satiety and reduces intestinal transit time and glycaemic response. It has been reported that weight loss is also related to a reduction in lipids or to an inhibitory effect on appetite [64,65].

The slight hypoglycaemic effect observed with the consumption of amaranth-enriched bread could be related to an effect of the amaranth’s components on insulin synthesis or on the peripheral use of glucose [64,65].

The data showed a simultaneous decrease in TC and, particularly, TG following consumption of amaranth-enriched breads, which is a relevant finding since this is not found with the administration of antihypercholesterolemic agents. These results were consistent with what was reported by Jeong et al. [66].

It was demonstrated that low TC and LDL-C concentrations and high concentrations of HDL-C reduce the risk of developing ischemic heart diseases [67]. In fact, the hypocholesterolemic effects of amaranth consumption could be associated with the lower atherogenic index and cardiac risk factor found in the treatments including *A. dubius*. Therefore, the consumption of amaranth-enriched bread could reduce the risk of cardiovascular diseases. However, it has recently been suggested that systemic inflammation, and not dietary or serum cholesterol, is the cause of chronic diseases [68,69]. In this regard, further research could address the question whether any specific *A. dubius* constituent leads to the reduction of inflammatory mediators.

Amaranth contains high concentrations of dietary fibre, predominantly insoluble fibre [24,63]. Several researchers have demonstrated that insoluble dietary fibre reduced cholesterol levels [42,70,71], as confirmed in the breads of the present trial containing amaranth. Wholemeal bread has been reported to make a positive contribution to improving the intestinal function and treating obesity, as it presents high contents of insoluble dietary fibre [72]. Therefore, amaranth-enriched bread contains an even higher content of this type of fibre due to the addition of amaranth. This favours both intestinal regularity and weight control. Moreover, previous research has reported a higher antioxidant activity and an increased content of total phenolic compounds in breads enriched with amaranth flour [73].

Considering that including amaranth flour at 200 g·kg^−1^ did not appear to produce superior results than including it at 100 g·kg^−1^ in the experimental diets, further research could be oriented to elucidate the optimum level of inclusion of *A. dubius* flour in breads.

## 5. Conclusions

The consumption of bread made with wholemeal amaranth flour could be associated with a better lipid profile as its diminished serum TG, TC, LDL-C and VLDL-C levels, as well as cardiac risk, and also increased the HDL-C. It is known that this pattern reduces the risk of heart attacks and/or high blood pressure. Amaranth-enriched bread could be used as a coadjuvant in blood glucose regulation and weight control due to its dietary fibre content. Bread with wholemeal amaranth flour, therefore, could be an alternative food which could help to both prevent and treat cardiovascular and metabolic diseases due to its hypoglycaemic and hypolipidemic effects.

## Figures and Tables

**Table 1:** Ingredients and proximate composition of breads made with amaranth flour, commercial bread and control diet (as feed, g·kg^−1^).

	Experimental Diets			
	CD (Ratarina^®^)	ABD10	ABD20	CBD
**Ingredients of breads**				
Bread	-	900.0	900.0	900.0
Oil	-	50.0	50.0	50.0
Vitamin–mineral premix ^†^	-	50.0	50.0	50.0
**Proximate composition**				
Dry matter	913.2	918.1	919.2	912.2
Ash (minerals)	78.1	20.0	31.4	13.2
Crude protein	262.0	183.7	189.2	155.8
Ether extract	21.3	70.0	86.9	10.4
Crude fibre	62.5	17.3	30.0	9.7
NFE ^‡^	489.3	627.1	581.7	723.1
TDN ^§^	-	806.2	803.2	790.1
Energy (kcal·kg^−1^)	3447	3942	3986	3648

CD: control diet. CBD: commercial bread diet. ABD10: diet bread with 100 g·kg^−1^ amaranth. ABD20: diet bread with 200 g·kg^−1^ amaranth. ^†^ Provided by Tecnología & Vitaminas S.L. (Alforja, Spain). Mineral and vitamins composition (g·kg^−1^ premix): Fe: 10, I: 0.2, Co: 0.02, Cu: 3, Mn: 10, Zn: 12, Se: 0.02, vitamin E: 3.2, vitamin B_1_: 0.2, vitamin B_2_: 0.6, vitamin B_6_: 0.2, vitamin B_12_: 0.002, calcium d-pantothenate: 2, Nicotinic acid: 4.4, choline chloride: 10, vitamin A: 1,800,000 IU, vitamin D_3_: 300,000 IU. ^‡^ NFE: nitrogen free extract. ^§^ TDN: total digestible nutrients.

**Table 2:** Serum biochemical profile and zoometric parameters in control rats and rats consuming sucrose water for 23 weeks.

	Control	Consuming Sucrose Water	95% CI	*p*
**Biochemical Parameters (mg·dL^−1^)**				
GLU	112.91	135.08	(−33.85, −10.49)	0.011
TC	100.77	133.90	(8.53, 57.73)	0.011
TG	144.30	260.13	(−163.47, −68.18)	<0.001
HDL-C	22.70	20.75	(−3.78, 7.68)	0.484
LDL-C	37.99	72.35	(16.65, 52.06)	<0.001
VLDL-C	51.77	28.77	(−32.52, −13.47)	<0.001
TC/HDL-C	5.56	6.09	(−0.93, 2.01)	0.455
TC/LDL-C	3.77	1.64	(−3.35, −0.91)	0.002
HDL-C/LDL-C	0.63	0.28	(−0.58, −0.11)	0.007
TP ^†^	7.86	7.52	(−0.07, 0.75)	0.011
**Zoometric Parameters**				
Body weight (g)	410.90	414.50	(−75.61, 98.99)	0.394
Body length (cm)	23.58	23.70	(−2.02, 2.12)	0.165
Abdominal circumference (cm)	20.28	20.27	(−2.84, 0.60)	0.572
Body Mass Index	0.74	0.74	(−0.24, 0.08)	0.356

^†^ Values expressed in g·dL^−1^. GLU: Glucose, TC: total cholesterol, TG: triglycerides, HDL-C: high-density lipoprotein, LDL-C: low-density lipoprotein, VLDL-C: very low-density lipoprotein, TP: total protein. CI: confidence interval for the difference between means.

**Table 3:** Effect of 8-week consumption of diets prepared with amaranth on abdominal circumference in rats (weeks 23 to 31 of age).

Diets	Sex	Abdominal Circumference (cm)							
		Before ^†^		*p*-Value	95% CI	After ^‡^		*p*-Value	95% CI
	**F**								
CD(N) vs. CD(E)		16.95	18.88	0.905	(−5.29, 1.44)	16.85	19.38	0.460	(−5.89, 0.84)
CD(N) vs. CBD(E)		16.95	17.63	0.999	(−4.04, 2.69)	16.85	18.13	0.999	(−4.64, 2.09)
CD(N) vs. ABD10(E)		16.95	17.13	0.999	(−3.54, 3.19)	16.85	13.25	0.022	(0.23, 6.97)
CD(N) vs. ABD20(E)		16.95	17.23	0.999	(−3.64, 3.09)	16.85	12.25	0.0004	(1.23, 7.97)
CD(E) vs. CBD(E)		18.88	17.63	0.999	(−2.12, 4.62)	19.38	18.13	0.999	(−2.13, 4.62)
CD(E) vs. ABD10(E)		18.88	17.13	0.963	(−1.62, 5.12)	19.38	13.25	<0.001	(2.76, 9.49)
CD(E) vs. ABD20(E)		18.88	17.23	0.981	(−1.72, 5.02)	19.38	12.25	<0.001	(3.76, 10.49)
CBD(E) vs. ABD10(E)		17.63	17.13	0.999	(−2.87, 3.87)	18.13	13.25	0.001	(1.51, 8.24)
CBD(E) vs. ABD20(E)		17.63	17.23	0.999	(−2.97, 3.77)	18.13	12.25	<0.001	(2.51, 9.24)
ABD10(E) vs. ABD20(E)		17.13	17.23	0.999	(−3.47, 3.27)	13.25	12.25	0.987	(−2.37, 4.37)
	**M**								
CD(N) vs. CD(E)		21.28	21.70	0.999	(−3.19, 3.54)	22.45	22.00	0.999	(−2.92, 3.82)
CD(N) vs. CBD(E)		21.28	21.28	0.999	(−2.77, 3.97)	22.45	21.28	0.999	(−2.19, 4.54)
CD(N) vs. ABD10(E)		21.28	21.40	0.998	(−2.89, 3.84)	22.45	17.33	<0.001	(1.76, 8.49)
CD(N) vs. ABD20(E)		21.28	22.78	0.998	(−4.27, 2.47)	22.45	16.13	<0.001	(2.96, 9.69)
CD(E) vs. CBD(E)		21.70	21.28	0.999	(−2.94, 3.79)	22.00	21.28	0.965	(−2.64, 4.09)
CD(E) vs. ABD10(E)		21.70	21.40	0.999	(−3.07, 3.66)	22.00	17.33	<0.001	(1.31, 8.04)
CD(E) vs. ABD20(E)		21.70	22.78	0.995	(−4.44, 2.29)	22.00	16.13	<0.001	(2.51, 9.24)
CBD(E) vs. ABD10(E)		21.28	21.40	0.999	(−3.49, 3.24)	21.28	17.33	0.006	(0.58, 7.32)
CBD(E) vs. ABD20(E)		21.28	22.78	0.995	(−4.87, 1.87)	21.28	16.13	<0.001	(1.78, 8.52)
ABD10(E) vs. ABD20(E)		21.40	22.78	0.999	(−4.74, 1.99)	17.33	16.13	0.999	(−2.17, 4.57)

^†^ Week 23; ^‡^ Week 31. CD: control diet, CBD: commercial bread diet, ABD10: diet bread with 100 g·kg^−1^ amaranth, ABD20: diet bread with 200 g·kg^−1^ amaranth. N and E: normal and elevated blood lipids and glucose levels, respectively. F: female. M: male. CI: confidence interval for the difference treatment between means. SEM = 0.6194. SEM = pooled standard error of the mean.

**Table 4:** Effect of 8-week consumption of diets prepared with bread of amaranth on serum glucose (GLU), total cholesterol (TC) and triglycerides (TG) in rats (weeks 23 to 31 of age).

Sex	Female				Male			
Diets	Means		*p*-Value	95% CI	Means		*p*-Value	95% CI
	**GLU** (mg·dL^−1^)							
CD(N) vs. CD(E)	124.13	163.20	0.092	(−81.14, 3.00)	119.63	174.10	0.003	(−96.55, −12.40)
CD(N) vs. CBD(E)	124.13	173.10	0.010	(−91.05, −6.90)	119.63	164.48	0.027	(−86.92, −2.78)
CD(N) vs. ABD10(E)	124.13	148.08	0.744	(−66.02, 18.12)	119.63	166.30	0.018	(−88.75, −4.60)
CD(N) vs. ABD20(E)	124.13	153.70	0.432	(−71.65, 12.50)	119.63	152.55	0.271	(−75.00, 9.15)
CD(E) vs. CBD(E)	163.20	173.10	0.999	(−51.97, 32,17)	174.10	164.48	0.999	(−32.45, 51.60)
CD(E) vs. ABD10(E)	163.20	148.08	0.989	(−32.57, 51.57)	174.10	166.30	0.999	(−34.27, 49.87)
CD(E) vs. ABD20(E)	163.20	153.70	0.999	(−31.42, 52.72)	174.10	152.55	0.854	(−20.52, 63.62)
CBD(E) vs. ABD10(E)	173.10	148.08	0.687	(−17.05, 67.10)	164.48	166.30	0.999	(−43.90, 40.25)
CBD(E) vs. ABD20(E)	173.10	153.70	0.925	(−22.67, 61.47)	164.48	152.55	0.999	(−30.15, 54.00)
ABD10(E) vs. ABD20(E)	148.08	173.10	0.998	(−47.70, 36.45)	166.30	152.55	0.995	(−28.32, 55.82)
SEM = 8.3651								
	**TC** (mg·dL^−1^)							
CD(N) vs. CD(E)	133.78	128.13	0.976	(−36.91, 48.21)	148.88	137.68	0.999	(−31.36, 53.76)
CD(N) vs. CBD(E)	133.78	117.80	0.984	(−26.58, 58.53)	148.88	115.23	0.257	(−8.91, 76.21)
CD(N) vs. ABD10(E)	133.78	108.20	0.673	(−16.98, 68.13)	148.88	100.23	0.013	(6.09, 91.21)
CD(N) vs. ABD20(E)	133.78	114.90	0.949	(−23.68, 61.11)	148.88	109.13	0.088	(−2.81, 82.31)
CD(E) vs. CBD(E)	128.13	117.80	0.998	(−51.97, 32.17)	137.68	115.23	0.827	(−20.11, 65.01)
CD(E) vs. ABD10(E)	128.13	108.20	0.917	(−32.57, 51.57)	137.68	100.23	0.136	(−5.11, 80.01)
CD(E) vs. ABD20(E)	128.13	114.90	0.997	(−31.42, 52.72)	137.68	109.13	0.506	(−14.01, 71.11)
CBD(E) vs. ABD10(E)	117.80	108.20	0.999	(−32.96, 52.16)	115.23	100.23	0.991	(−27.56, 57.56)
CBD(E) vs. ABD20(E)	117.80	114.90	0.999	(−39.66, 45.46)	115.23	109.13	0.999	(−36.46, 48.66)
ABD10(E) vs. ABD20(E)	108.20	114.90	0.999	(−49.26, 35.86)	100.23	109.13	0.999	(−51.46, 33.66)
SEM = 8.4617								
	**TG** (mg·dL^−1^)							
CD(N) vs. CD(E)	137.13	215.88	0.173	(−171.61, 14.11)	152.03	308.73	<0.001	(−249.56, −63.84)
CD(N) vs. CBD(E)	137.13	178.07	0.945	(−133.81, 51.91)	152.03	232.33	0.153	(−173.16, 12.56)
CD(N) vs. ABD10(E)	137.13	99.68	0.998	(−55.41, 130.31)	152.03	170.53	0.998	(−111.36, 74.36)
CD(N) vs. ABD20(E)	137.13	109.73	0.949	(−65.46, 120.26)	152.03	109.13	0.999	(−83.89, 101.84)
CD(E) vs. CBD(E)	215.88	178.07	0.969	(−55.06, 130.66)	308.73	143.05	0.207	(−16.46, 169.26)
CD(E) vs. ABD10(E)	215.88	99.68	0.004	(23.34, 209.06)	308.73	170.53	0.0003	(45.34, 231.06)
CD(E) vs. ABD20(E)	215.88	109.73	0.013	(13.29, 199.01)	308.73	143.05	<0.001	(72.81, 258.54)
CBD(E) vs. ABD10(E)	178.07	99.68	0.178	(−14.46, 171.26)	232.33	170.53	0.519	(−31.06, 154.66)
CBD(E) vs. ABD20(E)	178.07	109.73	0.360	(−24.51, 161.21)	232.33	109.13	0.050	(−3.59, 182.14)
ABD10(E) vs. ABD20(E)	99.68	109.73	0.999	(−102.91, 82.81)	170.53	109.13	0.998	(−65.39, 120.34)
SEM = 18.4642								

CD: control diet, CBD: commercial bread diet, ABD10: diet bread with 100 g·kg^−1^ amaranth, ABD20: diet bread with 200 g·kg^−1^ amaranth. N and E: normal and elevated blood lipids and glucose levels, respectively. CI: confidence interval for the difference between treatment means. SEM = pooled standard error of the mean.

**Table 5:** Effect of 8-week consumption of diets prepared with bread of amaranth on serum high-density lipoprotein (HDL-C), low-density lipoprotein (LDL-C) and very low-density lipoprotein (VLDL-C) in rats (weeks 23 to 31 of age).

Sex	Female				Male			
Diets	Means		*p*-Value	95% CI	Means		*p*-Value	95% CI
	**HDL-C** (mg·dL^−1^)							
CD(N) vs. CD(E)	22.76	18.21	0.999	(−16.13, 25.23)	19.70	22.99	0.979	(−23.96, 17.39)
CD(N) vs. CBD(E)	22.76	19.02	0.986	(−16.94, 24.42)	19.70	19.45	0.999	(−20.43, 20.93)
CD(N) vs. ABD10(E)	22.76	47.80	0.007	(−45.71, −4.36)	19.70	51.15	<0.001	(−52.13, −10.77)
CD(N) vs. ABD20(E)	22.76	47.75	0.007	(−45.66, −4.36)	19.70	41.00	0.038	(−41.98, −0.62)
CD(E) vs. CBD(E)	18.21	19.02	0.945	(−21.49, 19.87)	22.99	19.45	0.864	(−17.14, 24.21)
CD(E) vs. ABD10(E)	18.21	47.80	0.001	(−50.21, −8.91)	22.99	51.15	0.001	(−48.84, −7.49)
CD(E) vs. ABD20(E)	18.21	47.75	0.001	(−50.21, −8.86)	22.99	41.00	0.146	(−10.53, 30.83)
CBD(E) vs. ABD10(E)	19.02	47.80	0.001	(−49.45, −8.06)	19.45	51.15	<0.001	(−52.38, −11.02)
CBD(E) vs. ABD20(E)	19.02	47.75	0.001	(−49.40, −8.05)	19.45	41.00	0.034	(−42.23, −0.87)
ABD10(E) vs. ABD20(E)	47.80	47.75	0.999	(−20.63, 20.73)	51.15	41.00	0.887	(−10.53, 30.83)
SEM = 4.1113								
	**LDL-C** (mg·dL^−1^)							
CD(N) vs. CD(E)	82.58	66.74	0.998	(−38.84, 70.54)	98.60	52.94	0.190	(−9.03, 100.34)
CD(N) vs. CBD(E)	82.58	62.96	0.989	(−35.06, 74.31)	98.60	50.26	0.132	(−6.35, 103.34)
CD(N) vs. ABD10(E)	82.58	40.47	0.294	(−12.57, 96.81)	98.60	33.33	0.008	(10.57, 119.95)
CD(N) vs. ABD20(E)	82.58	45.21	0.477	(−17.31, 92.07)	98.60	39.14	0.024	(−2.81, 82.31)
CD(E) vs. CBD(E)	66.74	62.96	0.963	(−50.91, 58.46)	52.94	50.26	0.972	(−52.01, 57.37)
CD(E) vs. ABD10(E)	66.74	40.47	0.901	(−28.42, 80.96)	52.94	33.33	0.989	(−35.08, 74.30)
CD(E) vs. ABD20(E)	66.74	45.21	0.977	(−33.16, 76.22)	52.94	39.14	0.999	(−41.26, 68.11)
CBD(E) vs. ABD10(E)	62.96	40.47	0.967	(−32.19, 77.19)	50.26	33.33	0.997	(−37.76, 71.62)
CBD(E) vs. ABD20(E)	62.96	45.21	0.996	(−36.93, 72.45)	50.26	39.14	0.999	(−43.94, 65.43)
ABD10(E) vs. ABD20(E)	40.47	45.21	0.999	(−59.43, 49.95)	33.33	39.14	0.999	(−60.87, 48.50)
SEM = 10.8743								
	**VLDL-C** (mg·dL^−1^)							
CD(N) vs. CD(E)	27.43	43.18	0.186	(−34.54, 3.04)	30.41	61.75	<0.001	(−50.13, −12.55)
CD(N) vs. CBD(E)	27.43	35.62	0.949	(−26.98, 10.60)	30.41	46.47	0.178	(−34.85, 2.73)
CD(N) vs. ABD10(E)	27.43	20.00	0.976	(−11.37, 26.22)	30.41	33.11	0.999	(−21.49, 16.09)
CD(N) vs. ABD20(E)	27.43	21.95	0.999	(−13.31, 24.27)	30.41	28.61	0.997	(−17.00, 20.59)
CD(E) vs. CBD(E)	43.18	35.62	0.972	(−11.23, 26.35)	61.75	46.47	0.222	(−3.51, 34.07)
CD(E) vs. ABD10(E)	43.18	20.00	0.005	(4.38, 41.97)	61.75	33.11	<0.001	(9.85, 47.43)
CD(E) vs. ABD20(E)	43.18	21.95	0.015	(2.44, 40.02)	61.75	28.61	<0.001	(14.34, 51.93)
CBD(E) vs. ABD10(E)	35.62	20.00	0.196	(−3.18, 34.41)	46.47	33.11	0.414	(−5.43, 32.15)
CBD(E) vs. ABD20(E)	35.62	21.95	0.379	(−5.12, 32.46)	46.47	28.61	0.042	(−0.94, 36.65)
ABD10(E) vs. ABD20(E)	20.00	21.95	0.999	(−20.74, 16.85)	33.11	28.61	0.999	(−14.30, 23.29)
SEM = 3.7367								

CD: control diet, CBD: commercial bread diet, ABD10: diet bread with 100 g·kg^−1^ amaranth, ABD20: diet bread with 200 g·kg^−1^ amaranth. N and E: normal and elevated blood lipids and glucose levels, respectively. CI: confidence interval for the difference between treatment means. SEM = pooled standard error of the mean.

**Table 6:** Effect of 8-week consumption of diets prepared with bread of amaranth on serum cardiac risk factor (TC/HDL-C), atherogenic index ((TC – HDL-C)/HDL-C) and total protein (TP) in rats (weeks 23 to 31 of age).

Sex	Female				Male			
Diets	Means		*p*-Value	95% CI	Means		*p*-Value	95% CI
	**TC/HDL-C** (mg·dL^−1^)							
CD(N) vs. CD(E)	5.98	7.06	0.974	(−3.79, 1.63)	7.64	6.16	0.794	(−1.23, 4.18)
CD(N) vs. CBD(E)	5.98	6.60	0.999	(−3.32, 2.09)	7.64	5.99	0.650	(−1.05, 4.36)
CD(N) vs. ABD10(E)	5.98	2.50	0.003	(0.77, 6.19)	7.64	1.97	<0.001	(2.96, 8.37)
CD(N) vs. ABD20(E)	5.98	2.47	0.003	(0.81, 6.22)	7.64	2.89	<0.001	(2.05, 8.37)
CD(E) vs. CBD(E)	7.06	6.60	0.999	(−2.23, 3.17)	6.16	5.99	0.995	(−2.53, 2.88)
CD(E) vs. ABD10(E)	7.06	2.50	<0.001	(1.86, 7.27)	6.16	1.97	<0.001	(1.49, 6.90)
CD(E) vs. ABD20(E)	7.06	2.47	<0.001	(1.89, 7.30)	6.16	2.89	0.006	(0.57, 5.98)
CBD(E) vs. ABD10(E)	6.60	2.50	<0.001	(1.39, 6.80)	5.99	1.97	0.003	(1.31, 6.72)
CBD(E) vs. ABD20(E)	6.60	2.47	<0.001	(1.42, 6.83)	5.99	2.89	0.013	(0.39, 5.81)
ABD10(E) vs. ABD20(E)	2.50	2.47	0.999	(−2.67, 2.74)	1.97	2.89	0.994	(−3.62, 1.79)
SEM = 0.5380								
	**Atherogenic Index** (mg·dL^−1^)							
CD(N) vs. CD(E)	4.99	6.07	0.976	(−3.80, 1.64)	6.64	5.16	0.801	(−1.24, 4.19)
CD(N) vs. CBD(E)	4.99	5.61	0.999	(−3.35, 2.10)	6.64	4.99	0.660	(−1.07, 4.37)
CD(N) vs. ABD10(E)	4.99	1.50	0.003	(−0.77, 6.21)	6.64	0.97	<0.001	(2.94, 8.39)
CD(N) vs. ABD20(E)	4.99	1.52	0.003	(0.75, 6.19)	6.64	1.89	<0.001	(2.03, 7.47)
CD(E) vs. CBD(E)	6.07	5.61	0.999	(−2.27, 3.17)	5.16	4.99	0.998	(−2.54, 2.90)
CD(E) vs. ABD10(E)	6.07	1.50	<0.001	(1.84, 7.29)	5.16	0.97	<0.001	(−1.47, 6.91)
CD(E) vs. ABD20(E)	6.07	1.52	<0.001	(1.83, 7.27)	5.16	1.89	0.007	(0.56, 6.00)
CBD(E) vs. ABD10(E)	5.61	1.50	<0.001	(1.39, 6.83)	4.99	0.97	<0.001	(1.30, 6.74)
CBD(E) vs. ABD20(E)	5.61	1.52	<0.001	(1.37, 6.82)	4.99	1.89	0.013	(0.38, 5.82)
ABD10(E) vs. ABD20(E)	1.50	1.52	0.999	(−2.74, 2.70)	0.97	1.89	0.994	(−3.64, 1.80)
SEM = 0.5409								
	**TP** (mg·dL^−1^)							
CD(N) vs. CD(E)	7.65	7.27	0.998	(−0.51, 1.27)	6.90	6.91	0.190	(−0. 91, 0.88)
CD(N) vs. CBD(E)	7.65	6.86	0.989	(−0.10, 1.68)	6.90	6.51	0.132	(−0.50, 1.28)
CD(N) vs. ABD10(E)	7.65	7.10	0.294	(−0.35, 1.44)	6.90	6.49	0.008	(−0.48, 1.30)
CD(N) vs. ABD20(E)	7.65	6.31	0.477	(0.44, 2.23)	6.90	6.47	0.024	(−0.47, 1.32)
CD(E) vs. CBD(E)	7.27	6.86	0.998	(−0.48, 1.30)	6.91	6.51	0.994	(−0.49, 1.30)
CD(E) vs. ABD10(E)	7.27	7.10	0.901	(−0.73, 1.06)	6.91	6.49	0.989	(−0.47, 1.32)
CD(E) vs. ABD20(E)	7.27	6.31	0.977	(0.06, 1.85)	6.91	6.47	0.999	(−0.45, 1.33)
CBD(E) vs. ABD10(E)	6.86	7.10	0.999	(−1.14, 0.65)	6.51	6.49	0.999	(−0.87, 0.91)
CBD(E) vs. ABD20(E)	6.86	6.31	0.652	(−0.35, 1.44)	6.51	6.47	0.999	(−0.86, 0.93)
ABD10(E) vs. ABD20(E)	7.10	6.31	0.132	(−0.10, 1.68)	6.49	6.47	0.999	(−0.88, 0.91)
SEM = 0.1778								

CD: control diet, CBD: commercial bread diet, ABD10: diet bread with 100 g·kg^−1^ amaranth, ABD20: diet bread with 200 g·kg^−1^ amaranth. N and E: normal and elevated blood lipids and glucose levels, respectively. CI: confidence interval for the difference between treatment means. SEM = pooled standard error of the mean.
